# Intensive intervention for children and adolescents with autism in a community setting in Italy: a single-group longitudinal study

**DOI:** 10.1186/1753-2000-4-23

**Published:** 2010-09-01

**Authors:** Marco Valenti, Renato Cerbo, Francesco Masedu, Marco De Caris, Germana Sorge

**Affiliations:** 1 Department of Medicine and Public Health, University of L'Aquila, Italy; 2 Reference Regional Centre for Autism, Abruzzo Region Health System, L'Aquila, Italy; 3 The "Il Cireneo" Foundation for Autism, Italy

## Abstract

**Background:**

Previous studies have shown favourable results with intensive behavioural treatment for children with autism: evidence has emerged that treatment can be successfully implemented in a community setting and in adolescent participants. The aim of this study was to describe the 2-year adaptive functioning outcome of children and adolescents with autism treated intensively within the context of special autism centres, as well as to evaluate family satisfaction with the activity of the centres.

**Methods:**

Sixty participants with autism (20 females and 40 males, aged between 4 and 18 years) attending the semi-residential rehabilitation centres for autism located in the Abruzzo region (Central Italy) were followed up and their adaptive functioning was evaluated both at baseline and after one and two years using the Vineland Adaptive Behaviour Scales (VABS). Parents' satisfaction with the service was evaluated using the Orbetello Satisfaction Scale for Children and Adolescent Mental Health.

**Results:**

The increase in VABS scores was significant on several domains in the different gender and age categories. It is worth noting that male children had improved a great deal (roughly, an effect size >0.20) in the domains of communication, daily living and motor skills (effect sizes 0.34, 0.45 and 0.27 respectively) whereas in male adolescents, a notable increase in VABS scores was recorded in the domain of socialization only (effect size 0.23). On the other hand, adaptive behaviour in female children increased in the domains of socialization and motor skills (effect sizes 0.27 and 0.42 respectively) whereas in female adolescents, good results were achieved in the domains of daily living, socialization and motor skills (effect sizes 0.22, 0.26 and 0.20 respectively).

The level of satisfaction of users of the service over time was found to be substantial, even when they had recently started the program.

**Conclusions:**

Our results support the implementation of special autism treatment community centres, based on a parent co-directed rehabilitative, intensive and early intervention. Further experimental research designed to document the effectiveness of services provided to children and adolescents with autism in the community is recommended.

## Background

Autism spectrum disorders (ASD) are pervasive developmental disorders that dramatically impact on the lives of affected persons, their families and the broader community.

Prevalence estimates show a high degree of variation among studies; a recent overall random effects estimate of prevalence across studies of typical autism was 7.1 per 10,000 (95% CI 1.6 to 30.6) and of all ASD was 20.0 per 10,000 (95% CI 4.9 to 82.1) [1].

As reported in a recent review [2], research on interventions for autism mainly focuses on six topics: sensory integration and sensory-based interventions; relationship-based, interactive interventions; developmental skill-based programs; social cognitive skills training; parent-directed or parent-mediated approaches; and intensive behavioural interventions.

The efficacy of a range of different approaches, differing both theoretically and practically (educational, rehabilitative, cognitive-behavioural), is well documented but a clear superiority of one approach over another has not been shown [3-7]. All strategies however recognize the importance of an individualized approach. Therefore the intervention must be preceded by an accurate assessment of child's level of development and emerging skills in different areas, and must follow the hierarchy of cognitive processes. Treatment should also be as comprehensive, intensive and early as possible. Early intervention is fundamental to prevent the gradual isolation and the autistic deterioration of cognitive function and behaviour in general [8].

Previous studies have shown favourable results with early intensive behavioural treatment (EIBT) for children with autism [9]: a recent meta-analysis [10] demonstrated EIBT can be claimed to be an intervention capable of producing strong effects in children with autism. Although the expectation that 47 percent of youngsters who receive EIBT will reach normal developmental status is strongly questionable [11], evidence has emerged that EIBT can be successfully implemented in a community setting. Moreover, home-based EIBT and autism-specific nursery provision produce comparable outcomes in follow up, according to the pioneering findings of Lovaas [12]. On the other hand, the literature lacks sufficient evidence about the effects of intensive behavioural treatments in adolescents entering rehabilitation programs for the first time, so it remains important to replicate in adolescents the good findings obtained in children, particularly in community settings, which is the aim of this study.

Governments are increasingly mandating special autism services [13]. However, community mental health centres serve a low percentage of the children with ASD [14]. Moreover, it should be noted that mean age at diagnosis and subsequent access to treatment is greatly variable not only across countries, but also within the same country or region. Indeed in Italy, the geographical distribution of special autism centres is extremely variable and the presence of a specialist service for autism in Local Health Agencies (the district units of the National Health System) is nearly an exception rather than the rule. The unavailability of daily-accessible services in most areas of the country implies a delay between diagnosis and the beginning of the intervention: as a matter of fact, early intervention is not always the rule, and those affected are often taken in for treatment in late infancy or adolescence or not treated at all. Facing this scenario, the parents associations are playing a growing role in promoting local initiative to implement special autism treatment centres. A pioneering initiative has been implemented in the Abruzzo Region (central Italy), where autism centres were established following the interaction between a foundation of parents of persons with ASD and the local health agencies of the regional government. Here the collaboration between private social participants and the public health system also gave rise to regional guidelines for preventive, early diagnosis and treatment of persons with autism. This effort (also involving family paediatricians and school institutions of the Abruzzo Region), together with the enhancement of a centre for diagnosis and treatment of communicative-relational disorders (0-2 years) and the mandatory use of validated screening tools (such as the Checklist for Autism in Toddlers M-CHAT), have enabled, in 5 years, the reduction by half of the age at first diagnosis of autistic spectrum disorders in the Region. In fact, the average age of arrival of new cases diagnosed with ASD at the reference centres has fallen from 62 months (in 2001) to the current 34 months, providing an advantage in terms of potential development of language and intelligence in the children [15].

### Objective

The aim of this study was to describe the 2-year adaptive functioning outcome of participants with autism aged <18 years treated intensively within the context of the Abruzzo Region (Italy) special autism centres, as well as to evaluate family satisfaction with the activity of the centres after 2-years.

## Methods

### Design

The study was designed as a naturalistic longitudinal investigation. The study was uncontrolled. In fact, the absence of alternative rehabilitation options in the area represents a serious constraint to a controlled design with regard to ethical issues.

### Participants

The case-series consisted of 60 participants with ASD (20 females and 40 males, aged between 4 and 18 years) attending the semi-residential rehabilitation centres for autism located in L'Aquila, Lanciano and Vasto (the Abruzzo Region, Italy), and followed up from April 2007 (beginning of the program) to March 2009. Inclusion criteria were diagnosis of ASD and regular attendance at public school, which is compulsory in Italy until 16 years of age. In fact, the recruited case-series represents almost 65% of all participants officially registered in the catchment area with a diagnosis of ASD lower than 18 years of age. The admission to the centres depended only on the consent and willingness of participants' parents or tutors. No exclusion criteria were considered, as the program is by law open to all participants with ASD lower than 18 years. With reference to medically ascertained puberty, participants were classified as children (n = 26, 9 females and 17 males) or adolescents (n = 34, 11 females and 23 males).

All participants underwent standardized assessment using the ADOS - Autism Diagnostic Observation Schedule Italian version [16] whose modules are tailored for individuals that range in age from toddlerhood to adulthood, and customized for both nonverbal and verbal participants. In conjunction with the ADOS, the Italian version of the ADI-R Autism Diagnostic Interview - Revised [17] was also administered, allowing a parent or caregiver to answer a series of questions about the participant's behaviour. Diagnoses of ASD were assigned according to ADI-R and ADOS scores and to the ICD-10 criteria: diagnosis of autism included Pervasive Developmental Disorder N.O.S., but excluded Asperger's disorder. As intellectual disability and verbal ability can significantly impact the prognosis of individuals with ASD, both verbal and non verbal IQ were assessed using the Wechsler Intelligence Scale for Children - III (WISC-III) Italian version [18]. Psychometric testing of participants identified 43 out of 60 participants (71.6%) as having intellectual disability (full scale IQ <= 75): prevalence of intellectual disability was similar across gender (15 out of 20 females, 28 out of 40 males) and age categories (18 out of 26 children, 25 out of 34 adolescents).

Child participants had never taken psychoactive drugs. At time of admission into the centre, 12 adolescents were being treated with psychoactive drugs. All participants had not previously experienced intensive behavioural intervention.

All participants' parents (one or both as appropriate) gave informed consent for admission and treatment, as well as to the research use of data. Treatment was administered according to Helsinki declaration, rules of good clinical practice and ethics within the context of a public mental health service, and officially approved and authorised by the Local Health Agency authority.

### Setting and intervention

Treatment is based upon behaviour modification, though it cannot be regarded to as a pure ABA. Whereas in ABA the focus of treatment lies in the family/home, in our approach the main intervention activity is in the rehabilitation community: participants with autism are admitted to a specialised setting like the centre, where their abilities are explored and trained, and intervention rules are subsequently assigned to home and school. For each participant, skills to be increased and problem behaviours to be decreased are clearly defined in observable terms and measured carefully by direct observation, with independent verification by secondary observers. An initial assessment is conducted to determine skills that the learner does and does not have. The selection of treatment goals for each individual is guided by data from that initial assessment, and a curriculum inventory and sequence that lists skills in all domains (learning to learn, communication, social, academic, self-care, motor, play and leisure, etc.), broken into smaller component skills and sequenced developmentally, or from simple to complex. The overall goal is to help each learner develop skills that will enable him or her to be as independent and successful as possible in the long run. Behaviour change procedures are specified clearly. The instructions and prompts, reinforcers ("rewards") and materials used to develop each skill are tailored to the individual learner. There is a written program or set of instructions for teaching each skill; the behaviour analyst in charge of the programming trains everyone who works with the learner to implement those programs consistently. Work at home follows in sequence the centre's activities. It is particularly important for parents to be trained to implement the procedures outside of formal treatment sessions, in a variety of settings (home, playground, community); research has shown that otherwise, the learner's skills are not likely to generalize. Maladaptive behaviours (such as stereotypic behaviour, self injury, aggressive and disruptive behaviour) are explicitly not reinforced; appropriate alternative behaviours are taught and reinforced instead. Learner progress is measured frequently, using the direct observational measurement methods mentioned earlier. To display progress and organize tasks, graphical aids and sequential graphic agendas of work are used. Data are graphed to provide visual pictures of what is happening with each skill and each maladaptive behaviour targeted for treatment. The data are reviewed regularly by the behaviour analyst, directing the programming so that learning errors can be caught early and intervention methods adjusted promptly if progress is not satisfactory. Of course, depending on individual verbal ability, participants receive verbal instructions and are encouraged to engage in verbal communication. The behaviour analyst also observes treatment and provides feedback to those conducting interventions on an ongoing basis. Fundamental aspects of treatment are the "regularity" and "predictability" of the context within which the child's experiences are activated. The intervention is based upon consistency, stability and continuity of the attitudes of figures who relate to the child, in a pleasant relational manner that facilitates work on "joint attention" and, more importantly, the ability to use symbols for communication.

All participants regularly attend public school classes in the morning during the school-year, or alternative daily educational opportunities during the vacation months. The centre team in charge of the participants stays in close communication with the school team: school curricula are widely adapted to the specific characteristics of participants and the student's success is based on the fulfilment of objectives rather than grades. Every child who receives special education at school must have an Individualized Education Program (IEP), compulsory by law in Italy. The IEP has two general purposes: to set reasonable learning goals for a child, and to identify the services that the school district will provide for the child. The IEP is developed by a team of individuals that includes key school staff, centre team and the child's parents. The team meets, reviews the assessment information available about the child, and designs an educational program to address the child's educational needs that result from his or her disability. A child's IEP must also be reviewed at least annually thereafter to determine whether the annual goals are being achieved and must be revised as appropriate. The teaching method at school draws on the use of graphics and computer software, as well as regular physical activity and sport programs.

The centre intervention includes 3 hours of treatment daily (from 3 pm to 6 pm) 5 days a week, with groups of up to 20 participants separated according to age (children, teens). Two days per week include physical activity/sports programs.

Defining feature of the intervention is that the programs are directed by professionals with advanced formal training in behaviour analysis as well as supervised experience in designing and implementing behavioural programming for learners with autism and related disorders. The activity of the centres is supervised by a senior child and adolescent psychiatrist and a senior psychologist widely experienced in the treatment of participants with ASD.

### Measures

#### Assessment of Adaptive functioning

The Vineland Adaptive Behaviour Scales (VABS) survey form is a well-recognized instrument, with demonstrable reliability and validity both for individuals who are developing typically and those with disabilities [19]. It is also the most widely used measure for the assessment of adaptive functioning in children with autism [20]. Previous research has found that children with autism present a characteristic pattern of adaptive behaviour, as measured by the VABS (deficit in the domain of socialization, relative deficit in the domain of communication and relative strength in the domain of daily living) [21].The Italian form of the VABS was used in this study [22]. Four VABS skill domains were used in this study: Communication (receptive, expressive, and written language skills), Daily Living (personal self-care, domestic, and community living skills), Socialization (interpersonal, play or leisure, and coping skills) and Motor Skills (gross, fine). The VABS provides standard scores (*mean *= 100, *SD *= 15) and higher scores indicate better functioning. Scores on the VABS can range from four standard deviations below the mean to more than two standard deviations above the mean in a population with autism both with and without co-morbid mental retardation [23,24]. The importance of adaptive behaviour variability in autism is underscored by its strong prediction of prognosis [25]. Identifying sources of variability in adaptive behaviour is critical to obtaining a more complete picture of development in autism as well as identification of treatment targets [26]. All forms of the VABS can be used to measure the effectiveness of intervention strategies. VABS is a sensitive instrument for testing the effects of treatment on several outcomes of the autistic spectrum. In order to ensure higher reliability, the VABS were administered to each participant's parent by the same professional at the three scheduled times. The VABS norms used for comparison with the sample were those norms for disabled individuals.

#### Parents' satisfaction

The satisfaction expressed by the users (parents of participants) on the service is an unavoidable aspect of any accurate assessment of effectiveness and quality of a rehabilitative intervention [27,28]. Given the specificity of the setting, the parents' questionnaire of the *Orbetello Satisfaction Scale for child and adolescent mental health services *(OSS-cam) has been used, a tool validated for the Italian population [29] and considered as the gold standard for measuring users' satisfaction by the Italian Society for Child and Adolescent Psychiatry (SINPIA). As opposed to other established scales in the literature, such as the Parent Satisfaction Questionnaire [30], the OSS-cam scale analyses satisfaction with regard to aspects not directly depending on expectations. The measure consists of 46 items grouped into 7 sections: service accessibility, service environment, working style of operators, service organization, family participation, intervention outcome, final remarks. Likert-like scores for items range from 1 to 6 (ordinal scale: 1 = bad; 2 = poor; 3 = insufficient; 4 = sufficient; 5 = good; 6 = excellent) and the literature suggests that the number of score levels should not be lower than 5 or greater than 10 to maximise the discriminating power [31]. Scores for each domain range from 1 to 10 on an analogue scale.

The questionnaire was administered after 1 and 2 years from entry into the service.

### Statistical analysis

The intraclass correlation coefficient [32] was calculated as the reliability coefficient for each dimension of both the VABS and the OSS-cam scales.

An ANOVA for repeated measures according to a "one-within" design was performed [33], to answer the question of whether there is a change over time in VABS scores obtained at baseline, one and two years after intervention, with gender, considered as an exploratory variable, and age category (children, adolescents) as independent variables. The rationale for analysis by age groups stems from our interest in the applicability of intensive rehabilitation to participants entering the program in adolescence. In order to evaluate their clinical significance, findings were also interpreted in terms of effect size, comparing baseline vs. 2-year follow up data. Effect size values were calculated according to Hedges [34] as *(Y*_*2*_*-Y*_*1*_*)/s*_*p*_^*2 *^where Y_1 _= pre-treatment (baseline) mean, Y_2 _= post-treatment (2 years) mean, s_p_^2 ^= √{[(n_1_-1)s_1_^2 ^+ (n_2_-1)s_2_^2^]/n_1_+n_2_-2}, n_1 _= number of participants at pre treatment, n_2 _= number of participants at post treatment, s_1_^2 ^= pre-treatment variance, s_2_^2 ^= post-treatment variance.

The differences between OSS-cam scores at one and two years after interventions were evaluated using a Wilcoxon paired test.

Statistical significance was set at a type I error of 0.05

## Results

Table 1 shows the VABS scores at baseline, one and two years from the beginning of treatment. Table 2 shows VABS scores increase in terms of effect size. The increase of VABS scores is statistically significant on most domains in the different gender and age categories. As to clinical significance, evaluated in terms of effect size estimates, it is worth noting that male children improved a great deal (roughly, an effect size >0.20) in the domains of communication, daily living and motor skills (effect size 0.34, 0.45 and 0.27 respectively) whereas in male adolescents, a notable increase in VABS scores was recorded in the domain of socialization only (effect size 0.23). On the other hand, adaptive behaviour in female children increased in the domains of socialization and motor skills (effect size 0.27 and 0.42 respectively) whereas in female adolescents good results were achieved in the domains of daily living, socialization and motor skills (effect size 0.22, 0.26 and 0.20 respectively).

**Table 1:** VABS scores at baseline and after one and two years of treatment.

			FEMALES		MALES	
			
	TIME	ALL PARTICIPANTS n = 60 mean (sd)	CHILDREN n = 9 mean (sd)	ADOLESCENTS n = 11 mean (sd)	CHILDREN n = 17 mean (sd)	ADOLESCENTS n = 23 mean (sd)
**COMMUNICATION**	Baseline	78.88 (9.23)	79.67 (8.64)	72.59 (9.78)	75.34 (8.02)	84.18 (7.20)
	Year1	80.32; (9.37)	80.17 (8.25)	70.40 (7.97)	81.42 (8.71)	84.31 (7.75)
	Year2	84.00 (9.77)	84.06 (10.18)	73.23 (8.64)	87.02 (8.15)	87.93 (7.44)
						
**ANOVA REPEATED** **F; Prob > F**		**66.37; 0.0000**	**15.2; 0.002**	**7.32; 0.0041**	**124.16; 0.0000**	**68.41; 0.0000**
						
		**ICC = 0.92**				

**DAILY LIVING**	Baseline	78.7 (8.33)	78.22 (6.34)	80.77 (8.64)	75.05 (7.95)	80.66 (8.66)
	Year1	83.52 (8.98)	77.37 (6.71)	78.21 (9.27)	86.07 (8.15)	86.57 (8.26)
	Year2	87.04 (8.61)	77.46 (5.21)	87.08 (8.38)	89.87 (6.62)	88.67 (8.87)
						
**ANOVA REPEATED** **F; Prob > F**		**77.72; 0.0000**	**0.64; 0.5415**	**37.67; 0.0000**	**401.42; 0.0000**	**114.89; 0.0000**
						
		**ICC = 0.82**				

**SOCIALIZATION**	Baseline	72.89 (9.08)	62.50 (7.75)	68.18 (8.82)	77.45 (7.48)	75.84 (6.53)
	Year1	74.76 (9.23)	68.36 (6.41)	73.04 (8.99)	75.41 (10.65)	77.60 (8.20)
	Year2	79.20 (9.39)	68.86 (8.31)	75.60 (8.02)	81.59 (6.58)	83.20 (8.92)
						
**ANOVA REPEATED** **F; Prob > F**		**72.03; 0.0000**	**25.16; 0.0000**	**21.23; 0.0000**	**21.64; 0.0000**	**55.09; 0.0000**
						
		**ICC = 0.90**				

**MOTOR SKILLS**	Baseline	91.09 (11.26)	74.88 (8.39)	74.88 (8.39)	96.15 (7.01)	94.93 (9.57)
	Year1	93.94 (9.41)	84.07 (7.80)	84.07 (7.80)	95.62 (6.41)	99.41 (8.80)
	Year2	98.91 (10.25)	85.16 (6.37)	85.16 (6.37)	104.07 (7.74)	102.42 (8.39)
						
**ANOVA REPEATED** **F; Prob > F**		**136.15; 0.0000**	**126.92; 0.0000**	**29.54; 0.0000**	**175.47; 0.0000**	**85.49; 0.0000**
						
		**ICC = 0.94**				

P-values refer to ANOVA for repeated measures. ICC=intraclass correlation coefficient

**Table 2:** Effect-size values for VABS score change over 2 years of treatment

		FEMALES		MALES	
		
	ALL PARTICIPANTS n = 60	CHILDREN n = 9	ADOLESCENTS n = 11	CHILDREN n = 17	ADOLESCENTS n = 23
**COMMUNICATION**	0.07	0.06	0.02	0.35	0.11

**DAILY LIVING**	0.13	-0.04	0.22	0.45	0.19

**SOCIALIZATION**	0.09	0.27	0.26	0.13	0.23

**MOTOR SKILLS**	0.09	0.42	0.20	0.27	0.16

Table 3 shows how the level of satisfaction of service users is substantial: data clearly highlight that satisfaction remained quite constant over time with regard to all domains and items covered by the questionnaire: differences between 1-year and 2-year OSS-cam scores on the 7 domains are clearly not significant, thus indicating a continuing good feeling of participants' parents towards the service.

**Table 3:** Median values of the OSS-cam scale for parents' satisfaction.

Domains (score 1 to 10)	1 year	2 year	Wilcoxon paired test	ICC
**Items (score 1 to 6)**	**Median (1**^**st **^**- 3**^**rd **^**quartile)**	**Median (1**^**st **^**- 3**^**rd **^**quartile)**		

**Service accessibility**	8.0 (7.0 - 8.0)	8.0 (6.0 - 8.0)	p = 0.91	0.85
Route (distance, trip)	4.0 (4.0 - 5.0)	3.5 (3.0 - 4.5)		
Administrative procedures	5.0 (4.0 - 5.0)	5.0 (3.0 - 5.0)		
Parking facilities	5.0 (4.0 - 5.0)	5.0 (4.0 - 5.5)		
Waiting room (comfort, cleanness)	5.0 (5.0 - 5.0)	5.0 (4.5 - 5.0)		
Access for persons with disability	5.0 (4.0 - 5.0)	5.5 (4.0 - 5.5)		
Information about waiting lists	5.0 (4.0 - 5.0)	5.0 (4.5 - 5.0)		

**Service environment**	8.0 (7.0 - 9.0)	8.0 (7.0 - 9.5)	p = 0.90	0.81
Areas and furniture	5.0 (4.5 - 5.0)	5.0 (4.5 - 5.5)		
Playrooms and games	5.0 (4.0 - 5.0)	5.5 (4.0 - 5.5)		
Calmness and silence	5.0 (5.0 - 5.0)	4.0 (3.0 - 5.0)		
Materials and tools for treatment	6.0 (5.0 - 6.0)	6.0 (5.5 - 6.0)		
No smoking observance	5.0 (5.0 - 6.0)	6.0 (5.0 - 6.0)		
Cleanness	5.0 (5.0 - 5.0)	5.0 (5.0 - 5.5)		

**Working style of the operators**	9.0 (8.0 - 10.0)	9.0 (8.0 - 9.5)	p = 0.88	0.88
Simple language (no technical jargon)	6.0 (5.0 - 6.0)	6.0 (5.0 - 6.0)		
On time at appointments	5.0 (5.0 - 6.0)	5.0 (4.5 - 6.0)		
Client privacy	5.0 (5.0 - 6.0)	5.5 (5.0 - 6.0)		
Listening habits	6.0 (5.0 - 6.0)	5.5 (5.0 - 6.0)		
Expertise and professional skills	6.0 (5.0 - 6.0)	6.0 (5.0 - 6.0)		
Operator-participant relationship	6.0 (5.0 - 6.0)	6.0 (5.0 - 6.0)		
Kindness	6.0 (5.0 - 6.0)	5.0 (4.0 - 6.0)		

**Service organisation**	8.0 (7.0 - 9.0)	8.0 (7.0 - 9.0)	p = 0.95	0.86
Opening time	5.0 (5.0 - 5.0)	4.0 (4.0 - 5.0)		
Information about participant rights	5.0 (4.0 - 6.0)	5.0 (4.5 - 6.0)		
Team cooperation	5.0 (5.0 - 6.0)	4.5 (4.0 - 6.0)		
Support to the participant's school team	5.0 (5.0 - 6.0)	4.5 (4.5 - 5.5)		
Information exchange with other personnel	5.0 (4.0 - 6.0)	5.0 (4.0 - 6.0)		
Shortness of waiting times	5.0 (4.0 - 5.0)	5.0 (4.0 - 5.0)		

**Family participation**	8.0 (7.0 - 9.0)	8.5 (7.5 - 9.5)	p = 0.86	0.91
Information about the participant's clinical status	5.0 (4.5 - 5.5)	5.0 (4.5 - 5.5)		
Involvement in operators/school meetings	5.0 (5.0 - 6.0)	5.5 (5.0 - 6.0)		
Information about the intervention	5.0 (4.0 - 6.0)	5.0 (4.0 - 6.0)		
Involvement in operators/health system relations	5.0 (4.0 - 6.0)	5.0 (4.0 - 6.0)		
Feeling of having a say in the matter	5.0 (4.0 - 5.0)	5.5 (4.0 - 5.5)		
Information about prognosis	5.0 (4.0 - 6.0)	5.0 (4.0 - 6.0)		

Intervention outcome	8.0 (8.0 - 10.0)	8.0 (8.0 - 9.5)	p = 0.90	0.87
Service help to participant in facing daily problems	5.0 (5.0 - 6.0)	5.0 (5.0 - 5.5)		
Feeling confident about "what to do"	5.0 (5.0 - 6.0)	5.0 (5.0 - 6.0)		
Service help to participant's quality of life	5.0 (5.0 - 6.0)	5.0 (5.0 - 6.0)		
Feeling of not being alone	5.0 (5.0 - 6.0)	5.0 (4.5 - 6.0)		
Service help to family in coping with problems	5.0 (5.0 - 6.0)	4.5 (4.0 - 5.5)		

**Final remarks**				
(1) personal experience with the service	8.0 (8.0 - 10.0)	8.5 (8.0 - 10.0)	p = 0.96	0.88
(2) will suggest the service to other families	10.0 (9.0 - 10.0)	10.0 (9.0 - 10.0)	p = 0.92	0.81
(3) expectations have been fullfilled	9.0 (8.0 - 10.0)	8.5 (8.0 - 9.5)	p = 0.81	0.82

Scores for domains range from 1 to 10 (numeric ordinal scale). Scores for items range from 1 to 6 (ordinal scale: 1 = bad; 2 = poor; 3 = insufficient; 4 = sufficient; 5 = good; 6 = excellent.

## Discussion

This article presents findings from an outcome survey of the effects of intervention for children and adolescents with autism in a parent-mediated community setting in Italy.

Our overall data provide encouraging signs, though they are not conclusive, given the uncontrolled nature of the design of the study, about the effectiveness of the educational-rehabilitative intervention model 's ability to produce positive changes in participants' adaptive capabilities. As to the problems posed by the uncontrolled design, the absence of a control group has to be taken into account when considering the findings. We would underline that the participants' right to immediate intervention took priority over the ideal design, i.e. including random allocation to either an intervention or control group, but offering the control group the opportunity for intervention at the end of the study should it show positive effects. This study described a lengthy intervention in an area of the country with no alternatives available, so it would have been unethical to allocate participants to a control group, as delay in providing treatment could have had permanent deleterious effects on the functioning of the participants.

Evidence can be found in the literature that favourable prognostic changes occur without intervention during the follow up of participants with ASD, at least in high-IQ adolescents [35]. On the other hand, it is worth noting that 75% of the participants in the present intervention were lower-functioning. Moreover, follow up studies of children with autism have shown that aggravation of symptoms or deterioration in behaviour may occur in at least an half of children around the time of puberty and early adolescence [36]. This allows the findings obtained in our one-group study to be seen as potentially valid signs of an effective intervention, with the qualification that the controlled design remains the optimal choice.

Our data show that males achieved on average better results than females in the domain of communication: this finding indicates that future research might examine this potential difference more systematically. Moreover, female children had poor performance in the domain of daily living. We acknowledge that the small sample size here may have biased the results. Daily living and socialization are domains where achieving notable results depends not only by the treatment, but also by the extent and strength of social and family networks: our results confirm the necessity of holistic bases for any treatment in autism.

As to the communication domain, adolescents were better functioning than children at baseline, but could not improve over time to the same extent as the children. Adolescents have a longer learning history and history of negative reinforcement for certain communicative acts, leading to more successful escape from communicative demands, than do younger children.

Excellent results were recorded in the motor skills domain. The rehabilitation program includes two weekly physical activity sessions supervised by specialised professionals: following the literature, we strongly recommend an extensive practice of exercise programs within the context of long term rehabilitation intervention for autism [37].

A further potential limitation of the study lies with the contribution of parents to the assessment of participants' adaptive behaviour changes. As both parents and professionals contributed to the intervention, both are prone to bias (in either direction).

Results from parent satisfaction questionnaires showed a high degree of parental satisfaction with the treatment. According to the literature, the judgement about health outcomes is the most important predictor of the overall opinion on the quality of services. However, it is obvious that the analysis of satisfaction in the first two years of activity may provide only a broad illustration, as it is likely biased by a favourable effect related to the positive impact of new facilities opening in areas hitherto totally lacking institutional resources.

To meet the complex needs of people with autism, the Local Agency of the National Health System and the main association of parents of persons with ASD developed a new treatment system, according to subsidiarity principles: in other words a new approach (at least for the Italian context) to severe mental handicap, namely autism, which provides responsible and constructive cooperation between the various forces that interact with disabilities around the participant (i.e. the reference centre, the paediatrician, the school system and the family).

The intervention was also designed to involve family paediatricians and specialized diagnostic centres and to define a norm for suitable functional assessment, involving all the actors who work with the child. The drafting of an assessment protocol allowed for participants of various ages, functional levels and assistance needs. Daily care for the educational-rehabilitative treatment, which was conducted by a multidisciplinary team, with mixed public-private social management and the active participation of parents in managing the experimental project, has achieved results in both the degree of autonomy of children and teens and the satisfaction of parents users.

The family-professional collaboration was an essential element in the treatment and stemmed from the need to move from services centred on professionals to services focused on the family. In this model, professionals and families become partners in the project, enabling the sharing of responsibility and awareness of the objectives, as well as more generalization of skills, a larger emotional and social adjustment.

For every participant it is therefore necessary to have knowledge of different areas (family, school, social network) combining information obtained through direct observations of the child with those obtained from parents, to reach a clear framework of the participant which reveals strengths and weaknesses. The assessment is hence a bridge that leads from the diagnostic frame to the therapeutic contract, through a clinical pathway, allowing for continuity between the processes of diagnosis, evaluation, treatment and verification. An initial interview after the clinical diagnosis must, sometimes, lead to further medical examination to search for further etiopathogenic factors. Already at this initial stage, there is a need for psychological support and, sometimes, psycho-social assistance for parents to guide their choices regarding diagnostic as well as therapeutic needs. Additionally, after further careful evaluation with standardized tools, which are reliable and specific to autism, it can be provided with prognostic and therapeutic information to help decide on the overall treatment.

## Conclusions

Our results support the implementation of special autism treatment community centres, based on a parent co-directed intensive and early intervention. Further experimental research designed to document the effectiveness of services provided to children and adolescents with autism in the community is recommended.

## Competing interests

The authors declare that they have no financial competing interests. Costs of the intervention are fully covered by the Italian National Health System.

The first author (MV) is at the same time professor in a public university and parent of person with autism.

GS is the president of the "Il Cireneo" Parents Foundation for Autism in the Abruzzo Region (Italy).

## Authors' contributions

All authors read and approved the final version.

MV conceived the study, and participated in its design and coordination, and helped with the interpretation of the statistical analysis and drafting of the manuscript.

RC directed the rehabilitative intervention.

MDC designed and directed the psychological intervention and contributed to the assignment of VABS scores.

FM performed the statistical analysis.

GS participated in the study coordination and contributed to the assignment of OSS-cam scores.
